# Point-of-Care Blood Tests: Do Indian Villagers Have Cultural Objections?

**DOI:** 10.3389/fchem.2018.00505

**Published:** 2018-11-06

**Authors:** Marika Vicziany, Jaideep Hardikar

**Affiliations:** Faculty of Arts, National Centre for South Asian Studies, Monash Asia Institute, Monash University, Melbourne, VIC, Australia

**Keywords:** point-of-care blood testing, rural health in India, cultural obstacles, infrastructure obstacles, cost obstacles, benefits, needs of villagers

The key research question for this paper is whether Indian villagers would be resistant to the introduction of new technologies that could test blood in the villages where they reside, rather than at hospitals or clinics as is now the case. We have selected the subject of blood testing because screening and diagnosis of blood is relevant to many conditions that form part of India's disease burden—for example, anemia^1^, hepatitis and malaria. Point-of-care testing would involve collecting blood in minute quantities, namely via a few pinpricks, and testing it on site using simple methods that could be managed by people with low levels of literacy. The technology would provide a readout of the test results within minutes of the blood being applied to the testing medium. The collection of blood and the tests could be conducted in front of patients in their own homes and the blood would not require refrigeration, storage or transportation to urban hospitals or laboratories. In this paper we consider the possible obstacles to and benefits of point-of-care testing in Indian villages by examining the controversy surrounding rapid testing for TB in India. We then go on to discuss two possible kinds of obstacles that have been raised in discussions with colleagues in Australia and India, namely cultural perceptions of blood, and secondly the adequacy of village level health infrastructure^2^.

## The limits and benefits of point-of-care blood tests

Warnings have been issued about assuming that in India the technology of point-of-care diagnostics alone can solve the problem of giving access to the benefits of rapid test results without taking into account the “real world context … in which tests need to be scaled up” (Pai et al., 2012, p. 6). The context in which new technology is introduced needs to factor in complex considerations, not just resource constraints. Indeed, as Pai et al. noted, appropriate innovations need to be designed,

…from the ground up, to ensure that they are robust, field-tested in a variety of conditions, have built in capacities for reporting/notification, and are appropriately priced (Pai et al., 2012, p. 6.).

**Figure 1:**
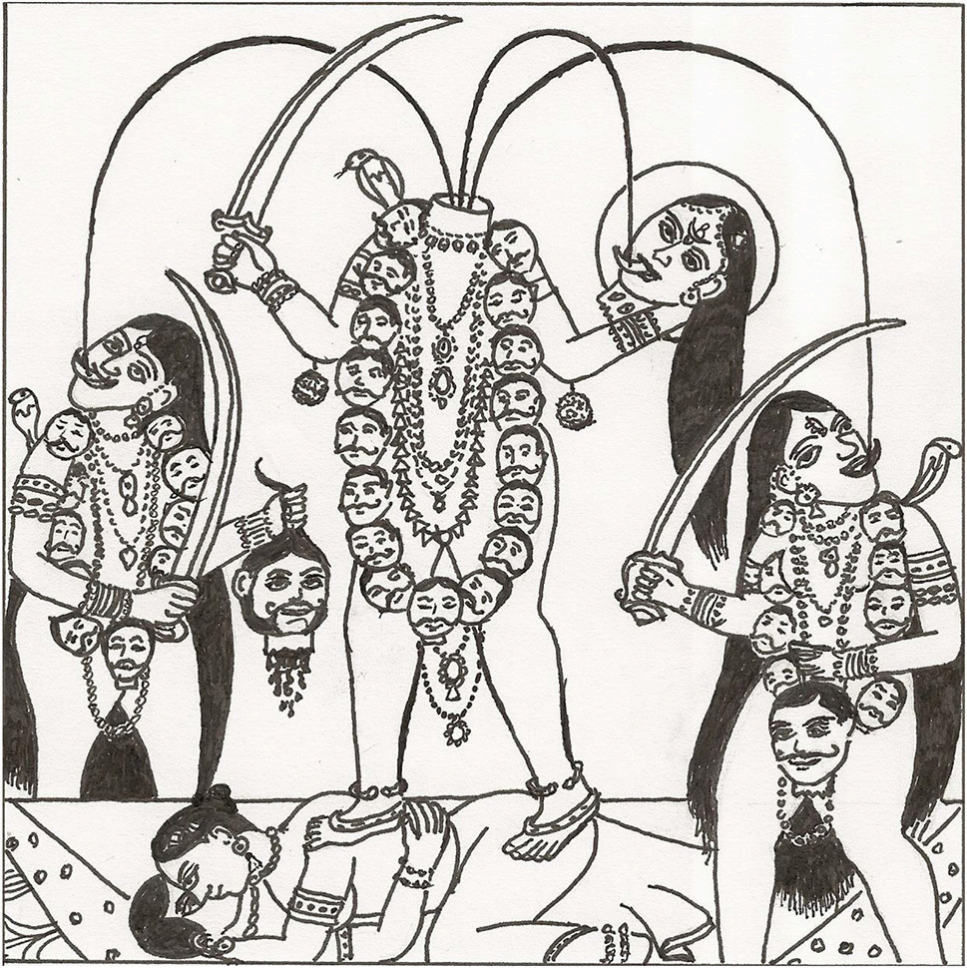
Image of Chinnamasta inspired by a typical poster (Drawn by John Harris for Marika Vicziany © Vicziany; Melbourne, March 2018).

The technology of conducting rapid tests for various illnesses in India's health system has widespread application, and the end-users of the technology should not be limited to health care workers in the villages. (Pai et al., 2012, p. 3. Figure 1) have identified five different groups of end-users in various settings: self-testing, testing in the community by health workers, testing in the clinic by healthcare providers, testing in laboratories, and testing of in-patients in hospitals. On the other hand, our focus in the second part of this paper on rural health care workers is justified because any new technology needs to win the support of the Indian Government and the existing healthcare workers in the government funded healthcare sector. More generally, Government of India approval is needed for any new technology, even if the private healthcare sector is the main end-user.

In 2012 rapid testing technologies gained a bad name in India when the government banned diagnostics for TB that used serological methods^3^ that had been popular with private sector practitioners but had been proven to be unreliable (Pai et al., 2012, p. 4; Jarosławski and Pai, 2012). The Indian government's decision was based on WHO recommendations and numerous independent assessments of the technology (Dinnes et al., 2007, p. iii), one of which estimated the large economic and social costs of the rapid TB tests delivering false negatives and false positives (Steingart et al., 2012, p. 695; Dowdy et al., 2011, p. 6). The evidence suggested that all new technologies for rapid testing need to be carefully screened and approved by the Government of India prior to being introduced. Ground level health care personnel also need to be involved in disseminating new technologies because it is sensible to use the existing health infrastructure that links villagers to the higher levels of care in hospitals. Moreover, local healthcare workers represent vested interests in the current system and it would be foolhardy to alienate them.

Despite these caveats, the benefits of point-of-care testing are considerable. The proposed testing equipment would be small scale, require no or minimal technical infrastructure and provide speedy results—under a minute in some cases. The rapid turnaround time between testing and results would also help doctors and other health professionals to provide rapid diagnoses, prescribe medicines, refer villagers to hospitals and more generally design long term treatment regimes in serious cases. Using conventional technologies that at present must be carried out in laboratories, makes turnaround times very long and in the process patients often fail to return to their doctors for further treatment (Pai et al., 2012, p. 2). To be effective, however, the health-medical system at all levels needs to stand behind point-of-care innovations. In particular, villagers need to be reassured about the tests' reliability and the best way of doing that is to demonstrate that the treatment prescribed by doctors is based on such rapid test results (Pai et al., 2012, p. 3).

Point-of-care diagnostics also has the potential to play an important role in dissuading sick villagers from dropping out of their existing treatment regimes. TB patients, for instance, are known for their on and off habits when taking drugs: when they feel ill they take the drugs, but stop taking them when they begin to feel better. Such temporary improvement does not contain the TB and, in fact, increases the risk of developing drug resistance to the disease. Readily accessible point-of-care diagnosis would help to demonstrate the ongoing presence of TB in such patients. Reminders by villagers, local healthcare workers or family members to the sufferers tend to be ineffective (Barnhoorn and Aixuaanse, 1992, p. 301). Point-of-care test results can serve as locally based evidence and these are likely to be more effective and less costly than external reminders involving home visits by doctors or hospitals sending out reminder postcards.

The social benefits of better methods for monitoring diseases are particularly pronounced in the case of conditions carrying social stigma. For example, the pressure to marry off daughters might encourage parents to hide information about illnesses in the family (Barnhoorn and Aixuaanse, 1992, p. 302; Harper, 2010, p. 208). Point-of-care diagnostics has the potential to reduce this kind of concealment by regularizing treatment and addressing the problem quickly. However, even the most compliant, drug abiding patient (see Venkat, 2017, pp. 101–103) might be compelled to abandon their treatment if they thought that their daughter's marriage was in jeopardy because of the shame and costs associated with medical intervention. Venkat's HIV infected patient asked to be killed because he believed that concealing his affliction was no guarantee of securing his daughter's future. We need to appreciate that point-of-care testing might be a valuable but insufficient response to how disease is entangled with family life and complex choices. This means that the need for a better understanding of local cultural and social conditions is even more compelling.

Despite the above extreme case, rapid testing for health conditions does hold out the prospect of improving the knowledge that patients have about their own medical conditions and prospects. Health professionals tend to assume that poor villagers, in particular, cannot understand their illnesses. This assumption induces an indifference amongst the health professionals with the result that patients often receive incomplete or even misleading information about the causes and cures for their illnesses (Barnhoorn and Aixuaanse, 1992, pp. 303–304). Even in relatively sophisticated South Asian cities like Karachi, one study reported that only 4% of the sample patients had been told that leprosy was caused by an infectious organism and could be eliminated by medication (Mull et al., 1989, pp. 799–807). Without this knowledge, or any warning of the short term side effects of treatment, patients were readily frightened into thinking that they have had an adverse reaction to the medication if suddenly their skin, urine or other bodily fluids turn black, green or orange (Mull et al., 1989, p. 807). If treatment is abandoned in such a scenario, there is a risk of the microbe becoming increasingly resistant to drugs (Mull et al., 1989, p. 808). Point-of-care diagnostics has the potential to place the evidence of ongoing illness before the patient in their own home, and such a simple and direct approach might also increase the villagers' sense that they are in charge of their own condition. Eventually, point-of-care testing can be administered by the patient themselves.

A particularly powerful example of how misinformation about medical technology is often promoted by well meaning, albeit impatient, health workers can be seen in the case of the blood donation campaigns of India. Motivators working for blood donation campaigns frequently oversimplify the nature of blood when stressing that in contrast to an organ donation, donating blood does not incur a permanent loss to the body. Such motivators define the average person as someone who has an “excess” of blood and so can benefit by getting rid of it (Copeman and Banerjee, 2017, Chapter 4)^4^. In other cases, motivators manipulate simplified Hindu concepts of pollution and purity. One New Delhi based blood bank director sought to persuade donors to get rid of their “polluted” or “senile blood” (Copeman, 2004, p. 128). In other cases, public blood donation campaigns are based on the opposite idea that blood is a scarce fluid and that donations are the equivalent of blood sacrifices^5^. Religious organizations in Indian cities have played a major part in promoting blood donations by emphasizing the good karma that will accumulate as a result of this kind of personal sacrifice (Copeman and Banerjee, 2017, Chapter 4). The problem with these contradictory justifications for donating blood is that none of them explains the nature of blood, why blood donations are important, and the relatively health low risks associated with giving blood^6^. Nor do these campaigns encourage people to have blood tests if they are ill.

By contrast, point-of-care testing has the potential to deliver multiple benefits to both urban and rural residents- in particular, better knowledge about the true nature of bodily fluids such as blood. This in turn, can contribute to improved medical information amongst the general population which might then be more inclined to donate blood which is in short supply. Finally, given the growing problem of drug resistance and the question of how we can be sure that a “cure” has been effective, point-of-care testing has the capacity to reduce the costs of the continuous monitoring that is needed to ensure that reinfection and relapses are addressed (Venkat, 2016, pp. 493–495).

Point-of-care testing is not limited to blood but we have chosen to focus on blood in the Indian situation because Monash University, where the authors are based, is developing a point-of-care technology of particular relevance to blood tests^7^. The authors are also India specialists but we believe that many of the issues that we address here are relevant to other countries and cultures. In the next section we analyse Indian perceptions of blood. In particular, we need to understand traditional values and understandings of blood and how, when and why they remain significant (Copeman, 2004, p. 128). Without this knowledge, widespread public support for new medical technologies in South Asia cannot be created. The anti-vaccination campaigns by militants in Pakistan have demonstrated the risks of alienating sections of the local community through misinformation and recourse to distorted religious ideology; health professionals and volunteers have been murdered because it was thought that polio vaccination was a Western conspiracy to sterilize people (BBC, 2018). Anti-polio health strategies in India have also been at risk because they “reflect the agendas of global funders, not the priorities of local communities” (Jeffery and Jeffery, 2011, p. 6.). Such warnings apply to the introduction of any new technology, including point-of-care testing.

## Part 1: are indian perceptions of blood an obstacle to blood testing?

### India's blood thirsty goddesses

The predominant image of blood in India is of sacrifices to Indian goddesses. These rituals typically take place in public areas such as in temples, town squares or villages. Such public displays are augmented by a popular visual art tradition that displays ferocious goddesses on posters, billboards and colored cloths that are sold on the streets of Indian towns and villages (see Figure 1). India has millions of bloodthirsty goddesses who demand respect; propitiation by worshippers calms the anger of a goddess and enables her to serve as the protector of the people who believe in her. The process of cooling the goddess's anger has typically involved blood sacrifices, in addition to prayers, offerings of alcohol and fruit, and the burning of incense. Depending on the scale of the perceived danger confronting a community or the wealth of the individual making the offering, the sacrificial animals are of large, medium or small size. The bigger the danger, the larger the sacrificial ceremony, which in the recent past might have involved thousands of animals (see Whitehead below). Hugh Urban explains the power of the popular buffalo sacrifice in the Kamakhya temple (Assam), “one of the most powerful centers of goddess worship in South Asia” (Urban, 2008, p. 500). In our own work on the Goddess Ekveera at her main temple on the Karle Hill near Lonavala (Maharashtra), we have seen hundreds of chicken offered for sale on the road approaching the temple (Vicziany et al., 2017). Prior to being sacrificed at the back of the shops, the chicken are presented to the demon who inhabits a shrine halfway up the hill. Until a few years ago, the sacrifices were conducted inside the temple but this has now been banned owing mainly to the vast increase in pilgrims and the lack of space inside the temple. In other parts of India, medium sized animals such as goats are preferred. Animal sacrifices are a way of pleasing the goddess and winning her intervention in curing family members from illnesses, protecting babies, promoting the fertility of brides and bringing good luck in the form of career promotions, pleasing exam results, cheaper mortgages and wealth.

Early European accounts of these rituals were preoccupied with the negative manifestations of religious worship (see below). New understandings, however, stress the positive association between hot, dangerous goddesses and feeding them with blood. The goddess Chinnamasta, for example, is typically depicted without her head which she holds in her left hand or on a plate (see Figure 1), while her right hand holds the sword with which she decapitated herself as an act of personal sacrifice. Three steams of blood gush from the base of her neck and typically she stands on an image of Kama (the god of love) copulating with his wife. In other depictions she stands on a lotus but both images are a metaphor for fertility and life. One stream of blood falls onto the outstretched tongue of the decapitated head of Chinnamasta^8^, while the other two streams feed the two attendants standing on either side. The blood of the goddess is imbibed as sacred food, a restatement of the basic theme of the never ending cycle of life, death and fertility. The goddess is ferocious, even to the extent of inflicting self-harm as a demonstration of her power, but the violence of the act is moderated by the life enhancing properties symbolized by the recycling of the blood, the most precious of all bodily fluids in Hinduism (see below).

The contemporary Hindu understanding of this image is that it celebrates the never ending cycle of life and death. Decapitation and the flow of blood back into the goddess is not only life affirming but it also asserts a superior form of existence that goes beyond materialism. In the word of one yoga website Chinnamasta has achieved that much sought after Hindu goal of reunion with the Supreme Being of the Universe.

This particularity (i.e., her headlessness) suggests her capacity of transcending the mind and its functions, so that in the end she achieves the ecstatic reabsorption in the Supreme Void of the Absolute Divine Consciousness (Chinnamasta, 2017a).

Another website includes various blogs by pilgrims asking the goddess to help them. “Please grant all my wishes” writes one devotee who addresses Chinnamasta as “maa” or mother (Chinnamasta, 2017b). We cite Chinnamasta here as only one, albeit dramatic, example of what it means to worship bloodthirsty goddesses.

The nurturing and protective side of India's mother goddesses explains their enduring attraction over the millennia. In the early twentieth century, Whitehead described the multiple functions of the blood sacrifices needed to cool the “hot” goddesses and win their support and protection. For example, dalit (former untouchable) servants walked around villages carrying the head of a sacrificed buffalo, rice and blood to make the village immune from evil spirits (Whitehead, 1988, 2nd edition)^9^; sacrificial food was distributed in shrines and urban streets to calm the smallpox goddess Mariamman; new wells, water tanks and tools were smeared with blood in order to prevent accidents; blood was mixed with grain and gram and on the fourth day whichever grain had sprouted was selected for the new year's crop; in Ellore, thousands of animals were killed for the goddess Mahalakshmi, with rich people offering between 20 and 30 animals; sacrificial blood was smeared on the doorposts of shrines to protect them; pieces of cloth dipped in blood were used as charms to protect cattle against disease; in Trichiniopolly 2,000 kids were sacrificed to ward off cholera and cattle plague; the temple priests, acting for the goddesses, drank blood in special rituals. Animal sacrifices were very expensive and care was taken to prevent the theft of soil stained by blood. In Whitehead's words, “…if any man from another village should take away and carry home even a small part of the blood, that village would get the benefit of the sacrifice” (Whitehead, 1988, p. 51).

In this last example, Whitehead was describing the buffalo sacrifice which, at the time he was writing and publishing his observations in 1921, was performed in Andhra Pradesh (“Telegu country”). In contrast to Hugh Urban's account of the buffalo sacrifice in Assam today, the villages in this area were not following any tantric rituals or temple based performance. Rather the ceremonies were carried out by dalit Malas and Madigas who had the task of killing the buffalo, first by bleeding and then cutting off the head and right foreleg. The goddess Peddamma or “Great Mother” had to be appeased because her anger had brought raging epidemics to the region. As a sign of the tremendous adulation of the villagers, the buffalo sacrifice was preceded by elaborate preparations that involved parading the buffalo through the village so that all residents could worship it; dressing the especially crafted clay image of the goddess; killing a ram and sprinkling the image with the ram's blood; carrying the image of the goddess to its final resting place under a canopy; placing cooked rice, turmeric and alcohol under the canopy; sacrificing a second ram; bringing more cooked rice and lamb from the village head's house to the canopy; spreading all the cooked food on margosa leaves before the image of the goddess; sacrificing a lamb and another ram. Finally the buffalo was prepared and sacrificed while all the surrounding villagers sang their praises to the goddess. On the second day, individual villagers brought their animals to be sacrificed by beheadings and more ceremonies (Whitehead, 1988, pp. 48–54). Whitehead's account represents the kind of elaborate village sacrifices that have today been replaced in most parts of India by more simple affairs involving fewer and smaller animals and sacrificial ceremonies that no longer require beheadings and bleedings.

Despite his invaluable account of village life, Whitehead's explanation for what he saw reflected the orientalist bias of his era. He insisted that the worshippers were fearful and not given to “praise and thanksgiving, no expression of gratitude or love, no desire for any spiritual or moral blessings” (Whitehead, 1988, p. 46). Contemporary scholars, by contrast, have a more rounded view of goddess worship and how fear converts into love because the “Great Mother” is a force that has a direct relevance to the daily lives of people. As Craddock notes, the followers of the smallpox goddess Mariyamman are considered blessed if they are infected with smallpox:

Mariyamman inflicts her devotees with pox, but the pox is a sign of her grace. Her devotees worship her out of love, not fear (Craddock, 1994, p. 5).

This love, the worshippers hope, will allow them to escape death. A few years ago, we witnessed a milder aspect of this mothering side in the case of the goddess Ekveera when one of the leaders (*patels*) of a Koli village in Mumbai city traveled for 4 h to present her with a personal invitation to his daughter's wedding. Only on completing this act could other guests be invited.

The animal sacrifices that remain common in urban and rural India today probably replaced much earlier human sacrifices^10^. According to Abbe Dubois, blood sacrifices in pre-Muslim India also involved human victims, but it was the “exclusive right of princes” to seek the support and protection of the goddess (Dubois, 1906, p. 647). Human sacrifices were governed by strict protocols, but had declined as a result of the influence of Muslim and European rulers of India who did not share the Hindu belief in propitiating goddesses^11^. Dubois' late eighteen century observations were widely cited by British officials who recorded their disgust with what they described as the degraded and licentious behavior tolerated at Indian temples and during festival times (‘Idolatry in India, 1830, pp. 87–118). While Hugh Urban notes that in Assam today it is said that rumors about secret human sacrifices to the goddess Kamakhya can be heard (Urban, 2008, pp. 501, 517–529), it is more typical for surrogates to be sacrificed instead of humans. For instance, ordeals that involve the loss of blood but do not lead to the death of the performer or worshipper continued into the late twentieth century. Hook swinging, for example, was the focus of a major campaign for its *continuation* in Kerala as late as 1987. The Elavoor temple committee and village devotees of the goddess Bhagavati challenged the State of Kerala and the police who banned the ritual that involved “copious flows of blood.”^12^. In most parts of India, however, melons, coconuts and other fruits are sliced rather than humans (Rodrigues, 2009, p. 271). Sometimes, substitute blood called *gurusi* is poured over altars as an alternative to human blood (Flood, 1997, pp. 183–184, 210–211). With the emergence of animal rights movements, a modern inversion of these processes has arisen with human blood donations being used as a substitute for animal blood in making offerings to the goddess (Copeman, 2008, p. 290). More broadly, gifts, alms or charities can be given by donors as ritual substitutes for sacrifice (Parry, 1986, p. 460). Biomedical donations of blood, organs and embryos are also now accepted as appropriate “sacrifices” (Copeman, 2011, pp. 1088–1089, 1091–1092). Hence the prominence of religious associations in the organization of blood donation campaigns (Copeman, 2005/2006, 2009).

How is the foregoing discussion about animal sacrifice, human sacrifice and blood relevant to our research questions about the attitude of Indian villagers to blood testing? As Flood has stressed, “Hinduism [and therefore India] cannot be understood without the goddess, for the goddess pervades it at all levels” (Flood, 1997, p. 196). Nor can India's bio-technological advances be understood without appreciating the importance of venerating the goddess, as the history of IVF demonstrates (Bharadwaj, 2006, pp. 453–457). Comprehending the cultural context for modern medicine, therefore, needs to begin with recognizing this elementary point. The goddess demands sacrifices characterized by violent and non-violent dimensions, thereby making sacrifice into one of the most powerful and fundamental ideas in India.

Moreover, in the villages of India, people are not squeamishness about matters of blood, because blood in its many forms is a familiar sight. Blood rituals are matters of public display and community festivals and they do not give rise to feelings of horror. Indeed, the opposite applies when sacrifices are intended to venerate the goddesses who protect believers from ill fortune, including illness and death. In the following section we shift our attention to human blood, specifically religious and secular Indian understandings of it. The values attached to human blood are not always on public display as in the case of animal sacrifices, but they impact on human behavior through socio-psychological processes.

### Hindu teachings about blood and semen

The preciousness of human blood is clearly stated in traditional Indian medical texts such as the *Sushruta Samhita* from the fourth century B.C.

Blood is the origin of the body. It is blood that maintains vitality. Blood is life. Hence it needs to be preserved with the greatest care (Bhishagratna, 1963, Vol. 1, pp. iii–iv).

What impact might such teachings have on the perceptions of villagers about the risks of giving blood for tests, for donations or receiving transfusions?

While the Sanskrit medical texts are not fully or accurately reflected in the medical practices of either elite Ayurveds^13^ or traditional healers in Indian villages today, many classical Hindu concepts have percolated down to the local level and to the population in general. Fieldwork amongst the villagers of Wardha District (Maharashtra) shows that some contemporary ideas about health^2^ can be related to classical Ayurvedic texts. Traditional ideas, however, do not necessarily pose obstacles to modern medicine. In the past, Ayurvedic practitioners did not hesitate to recommend bleeding as a cure for various illnesses; the removal of blood in this instance was totally acceptable provided it followed strict protocols including an assessment of the capacity of the patient to tolerate the loss of blood, the use of the proper instruments, the absence of clouds in the sky and the need to follow instructions about how surgical incisions were to be performed (Bhishagratna, 1963, Vol. 1, pp. 115–118). Thus we suggest that the removal of human blood for modern therapeutic purposes such as blood testing and blood donations does not contravene traditional Hindu medical ideas or practices. With point-of-care blood testing, the loss of blood is miniscule, but even with blood donations, where widespread reluctance to part with blood is often assumed because of tightly held traditional values, one reason given for not parting with blood by 40% of the 400 participants in one study was that “no-one ever asked them to give blood” (Dubey et al., 2014).

Yet Hindu understandings of blood are more complex than the above description suggests because Ayurvedic conceptions of life incorporate ideas from the much earlier Vedic period (c.1,500 B.C.E. onwards) in which blood is described as the “essence of the earthly body” (Doniger O'Flaherty, 1980, p. 19). This complexity needs to be appreciated to ensure that cultural obstacles to modern medical treatment do not emerge without warning. The Vedic ideas were restated in the *Sushruta Samhita* about 1,000 years later which gives a biochemical explanation of the role of blood in the body. Humans take in food, which is transformed in successive stages through the creative heat of the body. The first product is chyle, a white milky fluid produced by the intestine, and from that other organs and fluids are generated:

The chyle produces blood. From blood is formed flesh. From flesh originates fat which gives rise to bones. From bones originate marrow, which in its turn, germinates semen (Bhishagratna, 1963, Vol. 1, p. 108).

These seven products make up the *dhatus* or seven principles of the Ayurvedic human being. The blood of women, including menstrual blood, is created at the start of this process, whereas semen is the end product of a long process of refinement and concentration of “life energy.” The semen is concentrated in the head of men because “… this part [of the male body] above the neck belongs to God” (Carstairs, 1961, pp. 77, 78–79). As a result, the Hindu male must always have a well-groomed head because “The head is the root of a man's body–he is like a tree walking upside down” (Carstairs, 1961, p. 77). In many parts of contemporary India (and South Asia more generally) this notion of highly potent semen assumed mythical dimensions, such as in folk beliefs that one drop of male semen is made from 40 drops of blood that take 40 days to generate (Carstairs, 1961, pp. 77–79; Doniger O'Flaherty, 1980, p. 36). Despite this blood-semen model, we have found nothing in the classical Ayurvedic texts to show that these ideas will prevent Indian males from giving or receiving blood for medical emergencies. This does not mean, however, that Indian men are free of anxieties about losing blood. Rather that fear does not appear to be a direct outcome of the blood-semen model for male anxieties are focused on semen not blood (see below). Semen anxiety or *Dhat*, provides another good example of the importance of what Worthington and Gogne describe as the “culture-bound symptoms ….that need to be properly understood if satisfactory patient outcomes are to be achieved” (Worthington and Gogne, 2011, p. 1). The Indian cultural environment is not only defined by the kind of spiritual and traditional medical issues we have already discussed but also by hidden and deeply rooted psychological conditions (such as *dhat*) which we discuss next.

The Ayurvedic texts speak of the importance of preserving male semen to maintain health^14^. Misusing semen, for any purposes other than procreation, is condemned. Unwarranted semen loss can occur through improper behavior including the consumption of “hot” or inappropriate foods, inappropriate forms of sex including masturbation and contravening caste rules. In contemporary India, these traditional perceptions of human health give rise to anxiety in men of all ages who worry about, for example, night emissions—a primary symptom of *dhat* (Akhtar, 1988, pp. 70–71)^15^. Neurosis caused by fears of semen loss, are balanced out in traditional Indian medicine and folklore by the opposite but equally devastating condition of accumulating too much semen. Given the belief that semen is concentrated in the head of Hindu males, excessive amounts gives rise to mental disorders that can only be legitimately discharged by marriage. In Hinduism conjugal relations are an important part of the “householder” (*grhastha*) stage of a good Hindu life (*asrama*) where men and women marry, have children, work, enjoy material possessions, follow their caste norms, undertake good deeds and promote the productivity of home and society (Flood, 1997, pp. 61–66). The householder stage of life also obliges men and women to preserve their health and that of their children and in the process prolong life.

The second path to controlling unwanted quantities of semen and simultaneously conserving this precious *dhatus* is to become celibate (*sattiva*) and renounce everyday life and all of its distractions (*tapasya*) (Flood, 1997, pp. 88–90). Ascetics who take up this path of conserving semen (called *virya nirodh*) “accumulated special powers”^16^ such as levitation and being physically present in separate locations (Carstairs, 1961, p. 86). These special powers ultimately lead to enlightenment and the release of a *sannyasin* (renouncer) from the eternal cycle of rebirth. To bring this about, the ascetic must follow many rules, including the need to eat cool, calming foods to contain bodily desires (Srinivas, 2012, pp. 194–195; 197–200). More generally, “fierce psycho-physical austerities” are required (DeNapoli, 2009, p. 857) for by this means the renouncer is denying that reproduction triumphs over death (Steven Collins cited in Flood, 2004, p. 5). Exactly the opposite type of diet is needed by a male householder whose energy and masculinity is said to be promoted by eating hot foods.

Moreover, living the life of an ascetic is not merely about taking an accelerated path to individual merit and hence salvation (*moksha*) from the perpetual cycle of rebirth (*samsara*). Asceticism is also empowering at the social and political level as the life of Gandhi exemplified. His decision to adopt the most vigorous standards of celibacy in the 1940s was driven by night emissions of semen that he saw as a devastating weakness in his character. These human frailties, he believed, had reduced his political authority in the Indian struggle for independence and prevented him from putting an end to Hindu-Muslim killings (Lal, 2000, pp. 133–134). Indians today continue to have the highest respect for individuals who have chosen the life of an ascetic. Such perceptions have informed the wildly popular Satya Sai Movement and also given rise to many popular beliefs such as the idea that a wounded yogi bleeds semen rather than blood (Doniger O'Flaherty, 1980, p. 34). The implication in this last statement is that in a highly virtuous ascetic, semen has replaced blood.

So far we have argued that there appear to be no major cultural objections to human blood testing, donations or transfusions as far as traditional religious practices and medical texts are concerned. The respect that Indians have for bloodthirsty goddesses plays a role in the surviving traditional knowledge systems that protect villagers from ill fortune, but it has never prevented them from using other methods to improve their health. Hence, before the arrival of western medicine in India, there was an abundance of medical practitioners at the elite and popular levels, all borrowing with varying degrees of accuracy from the great Ayurvedic principles. There were also local medical traditions based on the knowledge accumulated by midwives, herbalists, soothsayers and spiritual leaders (Hardiman and Mukherji, 2012). Moreover, removing blood from the human body, as the *Sushruta Samhita* shows, has been a long accepted traditional medical practice. Finally, Hindu understandings of how the body works do not make blood and semen equivalents. The popular belief that'40 drops of blood make one drop of semen' makes the latter into the most refined of all human fluids while blood remains the most basic starting point for life - so basic that the blood of women is abhorred^17^. The available literature suggests that neurotic conditions or political concerns about excess amounts of semen or its loss are not directly connected to anxiety about blood.

## Part 2: health delivery systems in villages

Cultural considerations are not always pre-eminent in understanding the potential obstacles that face contemporary villagers in managing their physical or mental well-being The healthcare system often fails to deliver its promised benefits owing to “structural causes… having to do with how healthcare is accessed and delivered” (Worthington and Gogne, 2011, p. 1). What then is the situation of healthcare in rural India and how might that impact on introducing point-of-care diagnostics?

Indian governments began to focus on rural health within a few years of independence in 1947. The system that has evolved over time has resulted in a variety of programs to promote the health, nutrition and longevity of women and children in particular. In this section, we focus on the three main types of rural health workers: Auxiliary Nurse Midwives (ANMs), Anganwadi workers (AWWs) and Accredited Social Health Activists (ASHAs). The evolution of the rural healthcare system after independence in 1947 demonstrates that there is a functioning, albeit imperfect, local infrastructure that can be called upon to promote new medical technologies. We consider the essential features of this local system next: the establishment of the Auxiliary Nurse Midwives (1950s) to improve maternal healthcare in villages; the Anganwadi program (1975) to pay special attention to the health and nutrition of children aged between 6 months and 6 years of age; the Accredited Social Health Activists (2005) to ensure that every Indian village had one local health worker. The National Rural Health Mission (hereafter NRHM) of 2005 integrated all three types of health workers by setting up village level Health, Nutrition and Sanitation Committees (VHNSC) which became part of the self-governing structure of Indian villages. The committees had a wide brief and in addition to the health workers, they included the head (*pradhan*) of the village council (*panchayat*) acting as president, a secretary and representatives of local self-help groups (Sah et al., 2013, p. 114). Every village committee was also given an annual grant of Rs.10,000 for discretionary expenditures (called untied funds).

The committees have not worked well, largely because they are not taken seriously by village leaders. One study of Wardha District (Maharashtra) reported that the committee president was indifferent to the workings of the committee and that he and other members had no idea what they were supposed to do. Only the AWWs and ANMs knew about the program's objectives. As a result, untied funds were spent based on the decisions made by the ANM (Sah et al., 2013, pp. 114–115). Another study reported that the village presidents were only interested in receiving a commission for signing checks (Nandan et al., 2008–2009, pp. 31–34) even though the rules do not allow for such payments to be made. The checks release untied funds from government deposits in local banks. Government regulations require the village president's signature together with that of the ANM. If a president refuses to sign, then the banks cannot release the government funds, thereby depriving the whole village of its rightful share of assistance. The system of three layers of village level health workers has also been problematic: there have been problems internal to each of them in addition to the potential conflict between these overlapping levels of village based health programs. An assessment of each is given below.

### Auxiliary nurse midwives (ANMs)

The ANM system that began in the 1950s to provide maternal healthcare and midwifery services via sub-health centers in rural areas has changed. Instead of the initial functions, the ANM has emerged as a multi-purpose health worker with a primary focus on family planning and preventive healthcare, including immunization. The original duties of these government salaried workers, have increasingly become areas of health intervention for private sector institutions (Mavalankar and Vora, 2008, p. 7). Today the ANM has also become a commuter health worker rather than living in an allotted village. As a result government funded village healthcare has become less relevant and the health sub-centers themselves are poorly resourced. In the prosperous state of Gujarat, for example, one study reported that “55% of sub-centers do not have their own buildings and 78% do not have tap water” (Mavalankar and Vora, 2008, p. 10). Table 1 must, therefore, be seen as an ideal administrative structure rather than one that reflects realities on the ground. Despite these problems with the ANM system, important decision making responsibilities have fallen on her through the Village Health, Nutrition and Sanitation Committees because there is no one else to take up these tasks and also because the ANM's role in family planning reflects the long priority of Indian governments to focus on birth control (Vicziany, 1982, 1982-1983). That historical function has given the ANMs more authority than other village level health workers.

**Table 1:** Health Infrastructure of a Typical Indian District showing the Administrative Structure at the District, Block and Village Levels.

**Health infrastructure in a typical indian district**	**Population norm**	**Human resources available**
**DISTRICT LEVEL**		
District hospital	2–3 Million	Obstetricians, anesthetists, pathologists, pediatricians, general practitioners, nurses
First Referral Unit (FRU)	300,000–500,000	Obstetrician, general practitioners, nurses
**BLOCK LEVEL**		
Community Health Centre (CHC)	100,000–300,000	Any specialist, general practitioners, nurses
Primary Health Centre (PHC) (Old Block level)	100,000	General practitioners (2), nurses, LHVs, ANMs LHV, Lady Health Workers are in charge of 6 Health sub-centers.
PHC New level	30,000	General practitioner, nurse, LHV, ANM
Anganwadi Centre (reports to Block Development Officer)		AWW
**VILLAGE LEVEL**		
Sub-center	5,000	ANM
Village Level Functionaries	1,000	ASHA

*Adapted from Mavalankar and Vora (2008), p. 4, Table 1*.

*The authors have added the Anganwadis that report to the Ministry of Women & Child Development as part of the Integrated Child Development Services (ICDS) rather than the Ministry of Health and Family Welfare*.

### Anganwadi workers (AWWs)

The next layer of village level health assistance began in 1975 when the Indian government resolved to focus on the health and nutritional needs of under 6 year olds by setting up Anganwadi centers. The AWW is a local woman appointed to run such a crèche where children receive a morning snack and a cooked midday meal and have their weight checked monthly (as an indicator of nutritional status). They also provide mothers with basic medicines and advice about improving child health. A national assessment conducted in 2013–2014 reported that there were just over one million urban and rural centers in India with about 71 million children registered (PEO, 2015, p. 36). Between 4 and 5% of these children were severely malnourished and between 17 and 20% were moderately malnourished (PEO, 2015, p. 29O); on the other hand, about 77% of them had normal levels of nutrition. But the impact in rural areas was poorer because in 14 out of 18 Indian states the percentage of rural children in the normal nutritional category was significantly less than urban children (PEO, 2015, p. 33). Overall, the infrastructure of the Anganwadi centers was satisfactory but there were weaknesses in program implementation, namely the insufficient payments made to the AWWs, the infrequent visits by doctors to supervise them, the burdens of keeping 30 different kinds of records, and the lack of LPG gas for cooking (PEO, 2015, pp. 39–31, Dongre et al., 2008, p. 4). The lack of supervision by doctors was also noted in a study of a resettlement colony outside Delhi in 2015. In this case, although the AWWs had all completed 12 years of schooling, more than 80% did not know that the nutritional norms for the preparation of food had changed (Malik et al., 2015, pp. 202, 203–206).

Despite these problems the Anganwadi centers have had benefits beyond their priority focus on the health and nutrition of young children. One study reported that the centers had improved the pre-school education of children (Ade et al., 2010, pp. 541–546) and another that by functioning as day-care centers, they allowed mothers to take up extra paid work or to focus on heavy households duties such as fetching drinking water without having to worry about the safety of their children either at home or in the workplace (Dwivedi and Nagda, 2013).

### Accredited social health activists (ASHAs)

The ASHAs think of themselves as volunteers but many of them take these positions in the hope of switching to reliable jobs such as those performed by the Anganwadi workers. The ASHA is a female village resident who informs villagers about a range of health risks but her first responsibility is to encourage women to have their babies in hospitals. The ASHA receives an incentive payment of Rs 600 for every woman she refers for an “institutional birth.” However, a study from Wardha district, Maharashtra, noted that most ASHAs were not aware of this priority, while those who did know and were keen to earn incentive payments, could not do so. The report identified the Medical Officers in charge as the source of the problem—they withheld information about these incentives, thereby undermining the system (Gosavi et al., 2011, p. 36). Even when given, the financial incentives to ASHAs were too small to make a significant contribution to the ASHA's family income (Saprii et al., 2015, p. 95).

Despite these shortcomings, the NRHM has been credited with increasing the number of women giving birth in hospitals or clinics instead of families using traditional village midwives in the home. One reason is that the NRHM includes a cash payment of Rs. 1,400 to every mother in a deprived area who has an “institutional delivery” and who participates in the antenatal care programs (Vellakkal et al., 2017, p. 80). Vellakkal's study showed that the benefits of this initiative for the poor became more evident as the program was rolled out and that its relative success in rural areas favors continued public funding rather than the privatization of this aspect of public healthcare (Vellakkal et al., 2017, pp. 87–88).

While the healthcare infrastructure discussed above has many limitations, since 1950 publicly funded support to villages has nevertheless expanded, outreach to villagers has improved, discretional funds have been created and the range of services has diversified. The contributions to village health by semi-trained ASHAs, AWWs, and ANMS cannot be ignored. Moreover, it is easy to see where the system might be improved: for example, there is a close relationship between the efficient functioning of Angwandi centers and the proportion of AWWs who have studied beyond the Secondary School Leaving Certificate (SSLC) or Grade X at high school (Asha, 2014, p. 135, Table 4). Given high levels of undergraduate unemployment, India certainly has the human resources to upgrade the educational qualifications of village level workers if governments decide to do so.

### Government and private rural health services

In addition to the structure of government funded healthcare as set out in Table 1, the private health system has expanded rapidly. Table 2 shows that even in poor districts like Wardha in eastern Maharashtra, the number of private hospitals, dispensaries and maternity homes greatly exceeds those funded by the government. Moreover, unlike government institutions, the on-the-ground reality is likely to match the statistical information more closely because the people who pay for private medical treatment would not be willing to go to these institutions unless they had better services and facilities than those available free of charge in government institutions.

**Table 2:** Wardha District, Maharashtra Private and Public Health Infrastructure 2010–2011.

	**Primary health centers**	**Sub centers**	**Hospitals**	**Special hospitals**	**Dispensaries**	**Maternity homes**	**No. of beds**
Private	na	na	45	0	505	49	537
Public	27	181	10	1	36	24	933

*Statistical Information Centre, 2018*.

How does the existing rural health infrastructure relate to our question about the benefits of point-of-care blood testing within Indian villages? First, there is no doubt that India already has many semi-trained rural health workers throughout the country. Second, the simple and rapid blood testing technology we envisage would probably require even fewer skills and less training than what already exists. Third, there are additional options for delivering new technologies beyond the capacities of the current health administration; for instance, one trial in the mental health area involved recruiting female leaders of the local village cooperatives (*Mahila Mandals*). These women had much more local support than the ASHAs, AWWs, or ANMs because as representatives of the cooperatives, their personal interests meshed closely with those of the village community. Hence, the risk of these women leaving their villages to live in towns is low. Moreover, through the women it was also easier to respect local social and cultural norms and obtain the permission of the village *panchayat* (council) to undertake this trial (Jayaram et al., 2011, p. 263). When their work was opposed by a local, religious leader suffering from somatic delusions which he believed were punishment for his bad deeds, the women were able to explain that thanks to divine intervention, solutions to his problems could now be found through the new program started by the Maanasi Clinic (Jayaram et al., 2011, pp. 264–265).

## Conclusion

The key question we asked at the start of this paper was whether point-of-care blood testing in Indian villages was likely to encounter obstacles from Indian cultural attitudes or from the country's rural health infrastructure system. We have argued that it is essential for those bringing new technologies into rural India, to understand the cultural and social environment within which innovations will be functioning. As an example of our argument, we focused on Indian understandings of blood. These are complex and multifaceted, so we cannot pretend to have covered all aspects in this paper. Yet based on the history of animal and human sacrifice, the ongoing worship of blood thirsty goddesses and classical Ayurvedic medical teachings and practices, the likelihood of Hindu perceptions of blood constituting obstacles to point-of-care diagnostics is low.

We have also acknowledged that the relationship between blood and semen in the Ayurvedic texts and local beliefs systems represent powerful statements about the nature of male identity in India. However, the popular expression “40 drops of blood make one drop of semen” does not, despite superficial appearances, represent a relationship in which blood and semen are equivalents or where blood is more important than semen. As we explained, the original blood of the male body is transformed into semen by an elaborate biochemical process that separates these two substances and enables Hindu ideology to treat semen as the purest fluid of them all. Perhaps this is why Ayurvedic medical practice has no qualms about removing blood for therapeutic reasons? In our research on the psychological and physical pressure to conserve semen in the modern era—by ascetics, the practitioners of yoga, Gandhi or the sufferers from *dhat* syndrome—we found no mention of the need to conserve blood. *Dhat* syndrome or the fear of semen loss is well known in rural India yet we have no evidence to show that this anxiety flows over into anxieties about the loss of blood.

Possibly the largest obstacle to point-of-care blood testing has been India's experience with this some 6 years ago. As we noted at the start of this article, rapid tests for TB were fraught with difficulties, not because of any problems to do with Indian culture or health administration but rather because the newly introduced testing technology was unreliable and expensive, delivered false positives and false negatives, and yet was widely used by the private sector. Despite this, we have suggested that rapid blood tests for a variety of conditions at the point-of-care in rural areas have much potential and many advantages provided the Government of India supports and monitors such innovation.

Similarly, the rural healthcare system needs to stand behind the introduction of new, reliable, rapid testing methods. We described some of the important limitations in the administration of rural health; while these hamper the efficiency of the system, they do not render it ineffective. Point-of- care blood diagnosis is simple and easy to use and would not place additional burdens on local healthcare workers. At the same time, rapid testing methods may not be appropriate for all purposes - rapid analysis of blood types could, for instance, help identify urgently needed donors for particular blood groups, yet for other conditions such as TB, more time-consuming microscopy testing in laboratories may not be readily replaced. The specifics of each testing technology and the condition it seeks to address need to be identified and then set alongside the potential cultural, social, political and economic obstacles that the innovations might face on the ground. In another paper based on our fieldwork in Wardha district (Maharashtra) we report what villagers told us about the costs and benefits of modern medical interventions. The majority of the villagers said that point-of-care blood testing would be welcomed in their villages not only because it is rapid but also because it cuts down the costs of tests currently done in urban laboratories and hospitals. If celibacy and asceticism allow some particularly virtuous individuals to triumph over death through spiritual enlightenment, in our case study of Wardha district, villagers increasingly triumph over death by resorting to modern medical interventions^2^.

## Author contributions

MV (preferred name for publishing) has written some 15 books and over 120 peer reviewed book chapters and journal articles on India, China and Asian economic and political development since her doctorate from the School of Oriental and African Studies (University of London) in 1975. This paper is the first in a new project about rural health and politics in India, initiated by MV who is also the lead author of this paper. This new research builds on her earlier work about Indian health issues including papers about Indian blood banks, condom manufacture, HIV/Aids, family planning and the socio-economic problems faced by dalits (former untouchables) and tribal peoples, including food security. As the main author, MV identified the research focus, defined its parameters, analyzed the literature and wrote the text. JH is the second author whose contribution including checking the data, finding extra official documents and checking the text against his experience of Indian villages. JH is an independent journalist based in Nagpur, central India; he is also a member of MV's research team.

### Conflict of interest statement

The authors declare that the research was conducted in the absence of any commercial or financial relationships that could be construed as a potential conflict of interest. The handling editor declared a shared affiliation, though no other collaboration, with the authors at time of review.

## Footnotes

^1^Despite the shift in India's disease burden toward modern lifestyle conditions such as diabetes and heart disease, iron-deficiency anaemia still accounted for 11% of all of India's disease disability in 2016: (Indian Council of Medical Research et al., 2017), p.43.

^2^We have written a second paper about the perceptions and experiences of 36 villagers in Wardha District (Maharashtra State) with modern medicine and the role of cultural values, hospitalization and economic costs in shaping their opinions. In particular, we report on how the latter, namely economic costs, inform their views on point-of-care blood testing. For information about this forthcoming publication please write to marika.vicziany@monash.edu

^3^Seriologic tests measure the presence of antibodies in blood.

^4^We thank the authors for allowing us to read their forthcoming manuscript.

^5^Note this is based on urban samples e.g. the work of the Kolkata-based Association of Voluntary Blood Donors, West Bengal (AVBDWB).

^6^By comparison, Australian donors have access to comprehensive and reliable information about who is eligible to donate blood and the possible side effects. Some diminution of maximum vitality for a short period might be experienced although we know of one case where the loss of vitality lasted for two weeks. Normally, plasma donations are replenished by the body within 24 h while red blood cells take between six and eight weeks to rebuild. As a result blood donors in Australia can only give blood every three months. Australian Red Cross Blood Service (2018), http://www.donateblood.com.au/faq; http://www.donateblood.com.au/learn/donor-safety; http://www.donateblood.com.au/learn; http://www.donateblood.com.au/faq/age; http://www.donateblood.com.au/learn/what-happens (accessed 9 Jan. 2018).

^7^Other Australian universities are also developing rapid tests for various purposes. For example, we are grateful to Professor Robert Gibson, NHMRC Research Fellow and Director of the Foodplus Research Centre at the University of Adelaide, for sharing with us information about the dried blood spot technology that he and his team are developing to measure nutrients in blood (Email to Vicziany on 10 Dec. 2017).

^8^The analysis by Tull (2015) of the complex symbolism of the tongue of Kali also applies to Chinnamasta.

^9^The following examples are based on Whitehead (1988), pp. 46, 51, 65–67, 85, 92-93, 99, 109.

^10^During the reign of Queen Jijibai (d.1772) human sacrifices to the goddess Mahakali were common as a way of protecting the Panhala Fort (Maharashtra) from her enemies: “Parties of men scoured the country to procure human victims….” She allegedly gave land to one Teli (oil maker) in return for him agreeing to bury alive his daughter-in-law under one of the fort towers (Gazetteer of the Bombay Presidency Kolhapur (1886), pp.314-315). Flood also mentions an incident on the death of the last Mauryan king in 185 B.C.E. when his successor is said to have performed a human sacrifice in Kausambi (Flood, 1997, p. 52).

^11^The rules included sacrificing prisoners of war, never offering a Brahmin or Kshatriya for sacrifice, the physical perfection of the victim and the victim's innocence of any ‘serious crime'.

^12^For a discussion of this campaign and the work of J. H. Powell, Geoffrey Oddie and Nicolas Dirks on the subject of hook swinging in British India see Nair (2009), pp. 165–183.

^13^Ayurveds are medical practitioners who follow Ayurvedic principles: many of these today are trained in Ayurvedic colleges and have formal certificates but many more claim to be Ayurveds but lack formal training. The latter also borrow widely from all of India's many medical traditions in addition to borrowing ideas from western medicine. There has been a long running debate in India about how to stamp out these “quacks”.

^14^One drop of female milk is also equivalent to 40 drops of blood but very little has been written about the impact of this idea on female sexuality and identity—for example, DeNapoli (2009) does not deal with female semen.

^15^With the migration of South Asian peoples to the West, *Dhat* syndrome is a growing area of cross-cultural psychiatric treatment see Hamad et al. (2009), pp.64–65. On women suffering *dhat* see Avasthi et al., 2015, p. 519–520.

^16^For a discussion of the role of semen by the Rudolphs and Carstairs see Srinivas (2012), p. 192.

^17^The Vedic idea that women also produce semen that is concentrated in the vagina was replaced with an emphasis on sexually differentiated bodily fluids (Doniger O'Flaherty, 1980, pp. 32–33).
